# Removing Background Co-occurrences of Transcription Factor Binding Sites Greatly Improves the Prediction of Specific Transcription Factor Cooperations

**DOI:** 10.3389/fgene.2018.00189

**Published:** 2018-05-29

**Authors:** Cornelia Meckbach, Edgar Wingender, Mehmet Gültas

**Affiliations:** ^1^Institute of Bioinformatics, University Medical Center Göttingen, Georg-August-University Göttingen, Göttingen, Germany; ^2^Department of Breeding Informatics, Georg-August University Göttingen, Göttingen, Germany; ^3^Center for Integrated Breeding Research (CiBreed), Georg-August University Göttingen, Göttingen, Germany

**Keywords:** transcription factor (TF), TF cooperations, sequence-set specific TF cooperations, background correction, TF co-occurrences

## Abstract

Today, it is well-known that in eukaryotic cells the complex interplay of transcription factors (TFs) bound to the DNA of promoters and enhancers is the basis for precise and specific control of transcription. Computational methods have been developed for the identification of potentially cooperating TFs through the co-occurrence of their binding sites (TFBSs). One challenge of these methods is the differentiation of TFBS pairs that are specific for a given sequence set from those that are ubiquitously appearing, rendering the results highly dependent on the choice of a proper background set. Here, we present an extension of our previous PC-TraFF approach that estimates the background co-occurrence of any TF pair by preserving the (oligo-) nucleotide composition and, thus, the core of TFBSs in the sequences of interest. Applying our approach to a simulated data set with implanted TFBS pairs, we could successfully identify them as sequence-set specific under a variety of conditions. When we analyzed the gene expression data sets of five breast cancer associated subtypes, the number of overlapping pairs could be dramatically reduced in comparison to our previous approach. As a result, we could identify potentially cooperating transcriptional regulators that are characteristic for each of the five breast cancer subtypes. This indicates that our approach is able to discriminate specific potential TF cooperations against ubiquitously occurring combinations. The results obtained with our method may help to understand the genetic programs governing specific biological processes such as the development of different tumor types.

## 1. Introduction

Transcription factors (TFs) are a special class of cellular proteins that are essential for controlling different genetic programs such as adaption to the environment, immune response, organogenesis or embryonic development by regulating gene expression. The human genome encodes roughly 1500–2000 different TFs which bind to short degenerate DNA motifs, known as transcription factor binding sites (TFBSs). In higher organisms, the binding of TFs occurs in a specific combination within DNA regulatory regions (promoters as well as distal elements, such as enhancers) to form purposive dimers or higher order complexes to activate or repress their target genes. Due to the fact that eukaryotic DNA is packed in chromatin, TFs show additionally competing or cooperative DNA binding with chromatin associated proteins (Teif and Rippe, 2010). Besides this, based on the co-occurence of their TFBSs TFs exert functional cooperations which play an important role in the regulation of the different genetic programs in mammals (Boyer et al., 2005; Hu and Gallo, 2010; Neph et al., 2012). Today, it is well-known that the selection of cooperation partners for TFs depends on their biological functions, e.g., cell cycle control, cell homeostasis, or cell differentiation in different cell types. As a result of these properties, TFs change their partners to specify their functions according to the cellular context.

In the last decade, a various number of computational methods for the identification of cooperating TFs has been proposed (Hu et al., 2007; Van Loo and Marynen, 2009; Girgis and Ovcharenko, 2012; Ha et al., 2012; Sun et al., 2012; Deyneko et al., 2013; Nandi et al., 2013; Jankowski et al., 2014; Navarro et al., 2014; Meckbach et al., 2015; Wu and Lai, 2016; Spadafore et al., 2017). Among these methods, predicting the putative TFBSs in the sequences under study and building a meaningful quantification measure of the cooperation between two TFs are two essential steps to make the predictions successful. Based on these steps, different strategies/ideas have been used for the identification of cooperating TF pairs such as the TFBS co-occurrences of cooperative pairs are more often than expected by chance and have significantly closer distances. In this context, several methods such as statistical methods like the hypergeometric test, clustering approaches, randomized occurrence frequency model (OF_*r*_) or Markov models have been developed (Hu et al., 2007; Chuang et al., 2009; Girgis and Ovcharenko, 2012; Ha et al., 2012; Mysickova and Vingron, 2012; Sun et al., 2012; Nandi et al., 2013; Jankowski et al., 2014; Lai et al., 2014; Navarro et al., 2014; Spadafore et al., 2017).

Employing a comprehensive performance evaluation study on the prediction results of those methods, Lai et al. (2014) have shown that the success rates of different approaches strongly depend on the corresponding evaluation criteria. This finding is also supported by our results, which we have presented in Meckbach et al. (2015). However, the predictions of almost all of these methods suffer from many types of obstacles that might occur as a result of high background like common regulatory programs between cell types and the environmental components in their regulatory sequences like GC content or nucleotide composition - indicating the ratio of the constituent monomer units/bases- as well as the noise effect of false positive putative TFBSs. Hence, such obstacles lead into background co-occurrence of TFBSs and consequently the results of a certain method are often highly overlapping for different sequence sets. Zeidler et al. (2016) have clearly demonstrated this problem in their study for detection of stage-specific TF pairs in a time series data set during heart development. To overcome this problem, they have further applied Markov clustering algorithm (MCL) (Dongen, 2000) to the pairs predicted by MatrixCatch methodology (Deyneko et al., 2013). Although several negligible TF cooperations could be eliminated, the application of MCL algorithm in this context is only based on the observed frequencies of TFBSs and does not consider the sequence specific environmental components. Consequently, the results of this approach seem to be conservative and not sequence set specific, yet.

To deal with this problem to some extent, we applied in our previous study the average product correction (APC) theorem (Dunn et al., 2008) in order to determine for each TFBS pair their background co-occurrence resulting from their possibly false positive TFBS predictions in the entire sequence set under study. Although, with respect to APC theorem, the background noise effect of false positive TFBSs could be successfully eliminated in the detection of significant TF pairs, the power and functionality of APC theorem appears to be insufficient to handle the remaining obstacles for the identification of sequence-set specific TF cooperations. In order to overcome the missing point of PC-TraFF workflow (Meckbach et al., 2015), we propose in this study an efficient approach that accurately quantifies the level of background co-occurrence of two TFBSs considering different types of obstacles (mentioned above) in the sequences under study. For this purpose, by preserving the (oligo-) nucleotide composition of the sequences of interest, we create a sufficient number of new shuffled sequence sets and based on these sets the background co-occurrence of a TFBS pair is measured. This process ensures that TF cooperations, which are very sensitive regarding the context of nucleotides and the distance of their binding sites, will become remarkable small background-values in comparison to common (ubiquitously occurring) TF pairs. These ubiquitously occurring TF pairs are often found as significant for different sequence sets and are less susceptible to the behavior of their binding sites in the set of sequences. Consequently, removal of this background leads to the separation of sequence set-specific TF pairs from the common ones.

To demonstrate the performance and functionality of our proposed approach, we analyzed a simulation data set as well as five breast cancer subtype-associated gene sets, and present the results step by step by providing comparative analysis. These data sets have been chosen because the importance of cooperating TF pairs have been well-studied in Meckbach et al. (2015).

### Terminology

For the sake of simplicity, we adapt the terminology of our previous paper (Meckbach et al., 2015). In doing so, each match of a position weight matrix (PWM) with a segment of genomic DNA is called a (potential) *transcription factor binding site* (TFBS). TFBSs are represented by names of their corresponding PWMs. The PWMs of TRANSFAC (Wingender, 2008) used in this report are denoted with their TRANSFAC identifiers, the structure of which is: *V$factorname*_*version*, where “V$” indicates that the PWM is representing a TFBS of a vertebrate TF. *factorname* refers to the TF name, while there are more than one PWM representing the binding motif of a certain factor, *version* is required for the unambiguous identification of the PWM. TFBS pairs refer to co-occurring TFBSs. It is important to note that we cannot make any statement about the kind of interaction such co-occurrence may be associated with (cooperativity, synergistic or antagonistic interaction etc.). The term cooperation refers to any kind of functional cooperation and/or physical interaction between the constituents of the predicted TFBS pairs.

## 2. Results and discussion

In this study, we introduce an extension of our previous methodological approach PC-TraFF for the separation of sequence-set specific cooperating transcription factors based on the co-occurrence of their binding sites from common ones. The overall workflow of our approach comprises two parts. First, the original PC-TraFF algorithm is used in order to predict significant TFBS pairs in a set of sequence where PC-TraFF provides for each significant TFBS pair *t*_*a*_ and *t*_*b*_ a pointwise mutual information score ${\mathbb{P}𝕄𝕀}_{pc}^{APC}\left(t_{a};t_{b}\right)$. Thereby, the minimal and maximal distance threshold for two TFBSs to form a pair is set to 5 and 20 bp, respectively, in order to provide a proper comparison to the original PC-TraFF-results.

**Figure 1:**
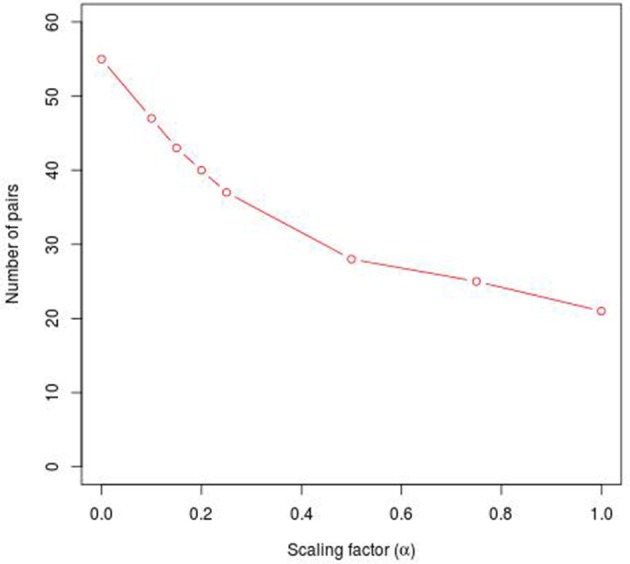
Number of specific TFBS pairs for the synthetic sequence set in dependence on different α-values. The synthetic sequence set consists of 200 sequences of length 1000 bps, each of these sequences contains artificially inserted binding site pairs (V$IRF1_01 - V$USF_01) for the cooperation between transcription factors IRF1 and USF1 with a minimal distance of 5 bp and a maximal distance of 20 bp. The α-value linearly influences the subtracted background level (e.g., α = 0 results in the subtraction of the *AVG*(ℙ𝕄𝕀(*t*_*a*_; *t*_*b*_)) value, α = 1 indicates the subtraction of the 2 × *AVG*(ℙ𝕄𝕀(*t*_*a*_; *t*_*b*_))-value).

Second, in order to separate PC-TraFF significant TFBS pairs into the two groups of sequence-set specific and common (generally important) combinations, we apply our extension approach. For this purpose, out of the sequences of interest, a sufficiently large number of background sets is created by shuffling the original sequences, whereby the general nucleotide composition of the sequences as well as the core of the putative TFBSs are maintained. For all these background sets, the original PC-TraFF algorithm is applied to calculate ${\mathbb{P}𝕄𝕀}_{pc}^{APC}$-values between all TFBS pairs. Afterwards, using these values the level of average background cooperation, which is defined as *AVG*(ℙ𝕄𝕀(*t*_*a*_; *t*_*b*_))-value, between two TFs based on their binding sites over all sets of background sequences is calculated. The subtraction of *AVG*(ℙ𝕄𝕀)-values from their initial ${\mathbb{P}𝕄𝕀}_{pc}^{APC}$-values results in the separation of sequence-set specific pairs from the common co-occurrences. To this end, we additionally introduced a factor α ∈ [−1, 1] to enlarge/reduce the effect of the subtracted background level by linearly influencing the subtracted average value *AVG*(ℙ𝕄𝕀(*t*_*a*_; *t*_*b*_)). If α = 1, the 2 × *AVG*(ℙ𝕄𝕀(*t*_*a*_; *t*_*b*_))-value is subtracted from the initiate ${\mathbb{P}𝕄𝕀}_{pc}^{APC}$-value, α = 0 results simply in the subtraction of the observed *AVG*(ℙ𝕄𝕀(*t*_*a*_; *t*_*b*_)) value, while an α-value of −1 results in the original PC-TraFF predictions. Thus, α enlarges/reduces the level of the subtracted background and is thereby influencing the number of identified specific pairs. However, our results suggest that the impact of α on the number of specific pairs strongly depends on the individual sequence sets and appears not to be linear (e.g., see Figure 1) although the factor itself has a linear influence on the subtracted background level.

It is important to note that the Results section of this study mainly considers the influence of our proposed extension approach on the cooperating TFs identified by the PC-TraFF algorithm. Researchers, who are interested in the biological functions of individual TF cooperations, are kindly referred to the original PC-TraFF paper (Meckbach et al., 2015).

### 2.1. Analysis of simulation data

Analyzing the sequences in the simulation data set, the original PC-TraFF algorithm identified 58 TFBS pairs as significant (α = −1), where the artificially inserted binding site pair of the cooperating transcription factors IRF1 and USF1 is on position 18 according to *z-score* ranking. However, applying our extension approach to the results of PC-TraFF, only three of the 58 significant pairs were determined as common ones (see Table 1) based on the calculated background co-occurence of TFBSs (α = 0). This rather low number of common pairs indicates that in a unspecific sequence set, the quantification of correct background could be difficult which, in the worst case, may cause that sequence-set specific cooperations cannot be separated from common ones. To overcome this problem, the consideration of the scaling factor α is important. Figure 1 shows the influence of α on the results. Although a variety of pairs are eliminated by means of different scaling factors, the inserted pair has been identified as sequence-set specific for each α-value. Considering the *z-score* ranking of TFBS pairs, the position of the inserted pair is rising with an increasing α-value (see Table 1). It has to be noted that the inserted binding sites are also matched by other PWMs, resulting in a variety of additional artificially arising TFBS pairs that consequently appear to be specific for the given sequence set.

**Table 1:** Total number of specific TFBS pairs for the simulation data set using different α-values.

**α-value**	**Rank of artificially inserted pair**	**Total number of pairs found**
α = −1	18	58
α = 0	16	55
α = 0.1	15	47
α = 0.15	14	43
α = 0.2	12	40
α = 0.25	11	37
α = 0.5	6	28
α = 0.75	6	25
α = 1	5	21

*The rank according to z-score indicates the position of the inserted pair. The scaling factor α = −1 indicates the significant TFBS pairs identified by the original PC-TraFF algorithm*.

### 2.2. Analysis of breast cancer subtype associated promoter sequences

**Figure 2:**
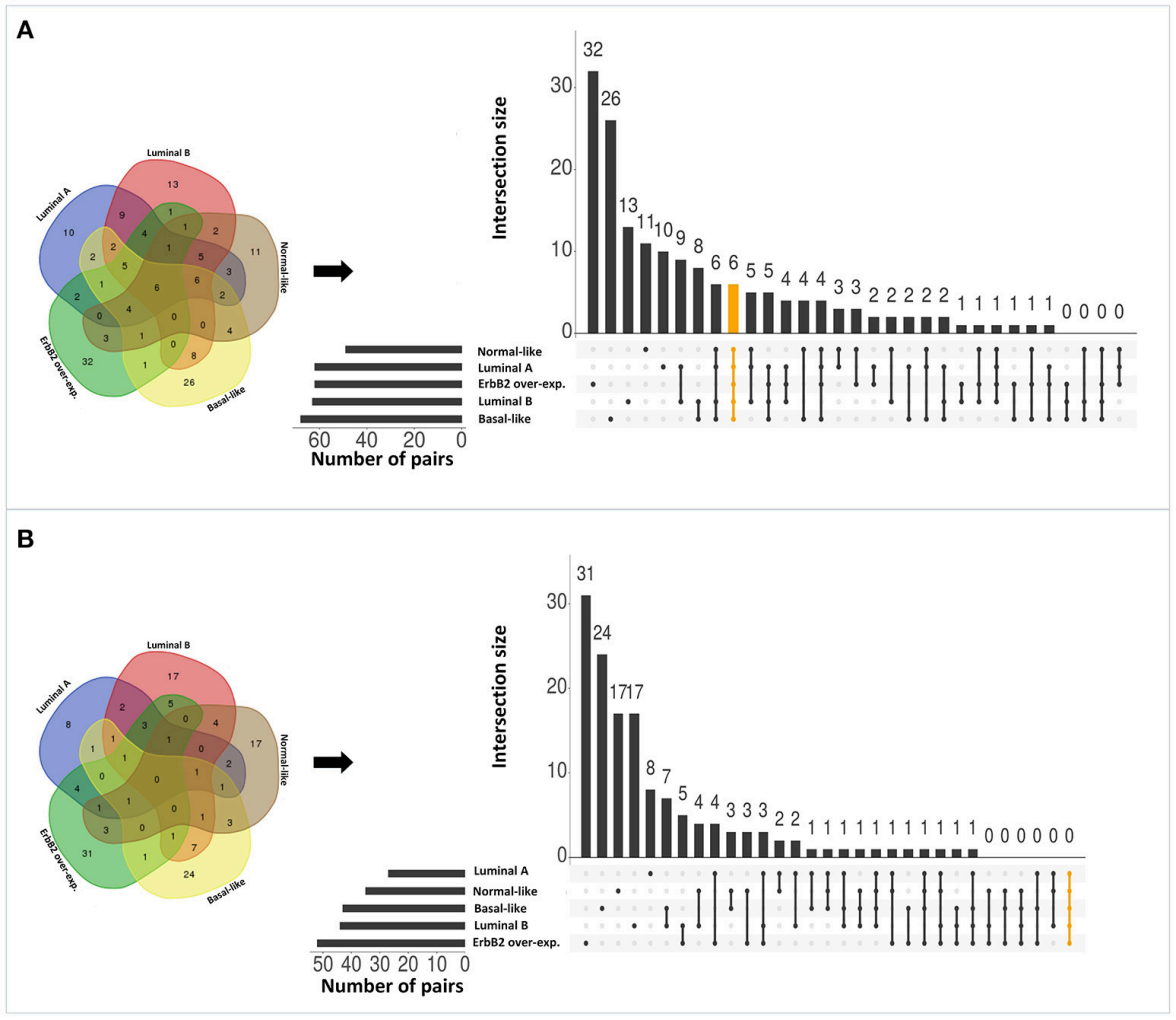
Number of significant TFBS pairs of five BRC-subtypes and their overlap represented in Venn diagrams and in matrix layouts using UpSet technique (Conway et al., 2017). Dark circles in the matrix layout indicate subtypes that are part on the intersection. Orange lines highlight the intersection between all BRC-subtypes. **(A)** Pairs identified by the original PC-TraFF version. **(B)** Sequence-set specific pairs determined by our extension approach using a scaling factor α = 0.2.

Applying the original PC-TraFF algorithm to each BRC-subtype associated promoter sequences, we observed: (i) 62 TFBS pairs for *Luminal A*; (ii) 63 pairs for *Luminal B*; (iii) 68 pairs for *Basal-like*; (iv) 49 pairs for *Normal-like*; and (v) 62 pairs for *ErbB2 over-expressing* data set as significant. A comparison between these pairs shows that there are several pairs found as significant for more than one BRC-subtype (see Figure 2A), although the promoter sequences in all subtypes are unique (not overlapping). The reason of these overlapping pairs could be due to the same origin of the data and common regulatory programs which interfere with the identification of BRC-subtype specific TF cooperations.

**Figure 3:**
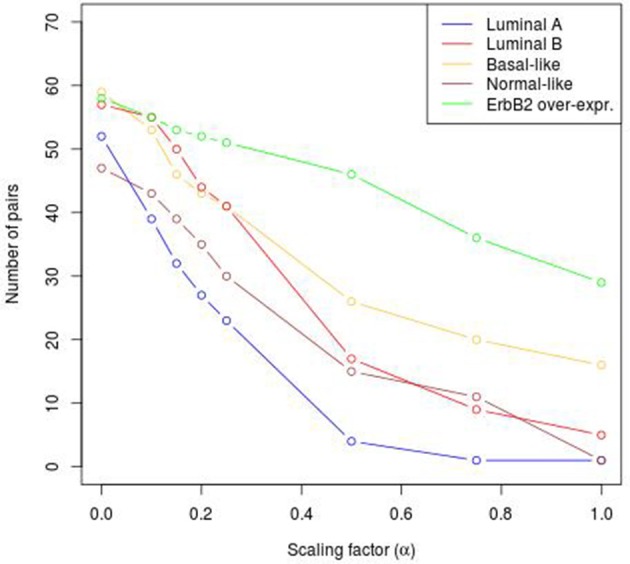
Number of sequence-set specific pairs found in the promoter sequences of differentially expressed genes of five BRC-subtypes depending on the α-value. The α-value linearly influences the subtracted background level (e.g., α = 0 results in the subtraction of the mean, α = 1 indicates the subtraction of the 2 × *AVG*(ℙ𝕄𝕀(*t*_*a*_; *t*_*b*_))-value).

To reveal the BRC-subtype specific TF cooperations, we additionally applied our extension approach using different α-values to these significant pairs. The results of this analysis indicate that the scaling factor α dramatically influences the number of sequence-set specific TFBS pairs. For example, on average 90% of the significant pairs have been determined as sequence-set specific by setting α = 0, and 66% or 35% of significant pairs are assigned as sequence-set specific by setting α = 0.2 or α = 0.5, respectively (Figure 3). Further, Figure 3 shows that, the influence of the scaling factor α is not consistent between the different sequence sets. While the number of specific TFBS pairs detected for *Luminal A* promoter sequences is dramatically decreasing and finally, 1% of all significant pairs have been determined as specific, the number of specific pairs for *ErbB2 over-expressing* promoter sequences has only slightly decreased in accordance with the increment of α-value and in an extreme case (α = 1) 47% of significant pairs in this subtype are assigned as specific. In addition, Figure 2 depicts in detail for α = 0.2 the differences between significant and specific pairs for any BRC-subtype. By considering the sequence-set specific pairs, it is remarkable that like in the original PC-TraFF analysis, the *Luminal A* promoter sequence set has the lowest number of unique pairs (eight), and *ErbB2 over-expressing* promoter sequences have the largest number of unique TFBS pairs. The intersection of all BRC-subtypes specific pairs is zero.

Interestingly, after applying our extension approach, there are more sequence-set specific unique pairs for *Normal-like* and *Luminal B* subtypes (Figure 2B) than significant unique pairs (Figure 2A). For *Normal-like* data set, there are 11 significant and 17 specific unique pairs. In particular, six pairs that were identified in the original PC-TraFF analysis for several subtypes are determined to be solely sequence-set specific for *Normal-like* subtype. For example, the pairs (V$CEBP_02 – V$HMGIY_Q6) and (V$ELK1_02 – V$CETS1P54_01) are significant for four different breast cancer subtypes or the pair (V$CEBPB_02 – V$CEBP_Q2) is significant in the originial PC-TraFF version for three BRC-subtypes, but they are sequence-set specific only for *Normal-like* subtype (for details see Table 2).

**Table 2:** Pairs that were identified as significant by PC-TraFF algorithm (α = −1) for different BRC-subtypes but are specific solely for a certain subtype using an α-value of 0.2 for the background correction.

**Specific for subtype**	**TFBS pairs**	**Significant in subtypes**
Normal-like	V$CEBPB_02 - V$HMGIY_Q6	*Basal-like, Luminal A, Luminal B, Normal-like*
	V$ELK1_02 - V$CETS1P54_01	*Basal-like, Luminal A, Luminal B, Normal-like*
	V$CEBPB_02 - V$CEBP_Q2	*ErbB2 over-expressing, Luminal B, Normal-like*
	V$NFKB_Q6 - V$SP1_Q4_01	*Luminal A, Normal-like*
	V$EGR_Q6 - V$AHRHIF_Q6	*Basal-like, Normal-like*
	V$GR_Q6_01 - V$PR_Q2	*ErbB2 over-expressing, Normal-like*
Luminal B	V$CETS1P54_01 - V$AHRHIF_Q6	*Luminal A, Luminal B, Normal-like*
	V$E2F_Q3_01 - V$PEBP_Q6	*Luminal A, Luminal B*
	V$MYCMAX_B - V$AHRHIF_Q6	*Basal-like, Luminal A, Luminal B*
	V$NFKB_Q6 -V$E2F_Q3_01	*Luminal A, Luminal B*
	V$NFKB_Q6 -V$AHRHIF_Q6	*Luminal A, Luminal B*
	V$CETS1P54_01- V$CP2_02	*Luminal A, Luminal B*
	V$CETS1P54_01 -V$MYCMAX_B	*Basal-like, Luminal A, Luminal B, Normal-like*

For *Luminal B* subtype, 13 pairs were uniquely identified as significant by the original PC-TraFF algorithm and 17 pairs were uniquely assigned as specific. In this case, seven pairs that were common in the original PC-TraFF analysis have been determined to be sequence-set specific only for *Luminal B* subtype. Further, three of the unique significant pairs (V$MYB_Q5_01 – V$MAF_Q6_01, V$NFKB_Q6 – V$CP2_02, V$HMGIY_Q6 – V$MAF_Q6_01) were assigned as common co-occurences according their negative ℙ𝕄𝕀^*specific*^-values.

Besides this, there are further six pairs identified by the original PC-TraFF algorithm as significant for all five BRC-subtypes, but they are assigned to be specific only for some of these subtypes (for details see Figure 2 and Table 3). For example the TFBS pair (V$CEBPB_02 – V$STAT6_01) indicating the cooperation between the transcription factors CEBPB and STAT6 can still be found in the sequence-set specific pairs of *Luminal A, Luminal B* and *Basal-like* subtypes. In contrast, the pairs (V$MYCMAX_B – V$E2F_Q3_01) and (V$STAT6_01 – V$HMGIY_Q6) have been determined as specific only for *Basal-like* and *Normal-like* promoter sequence sets, respectively.

**Table 3:** TFBS pairs, which were identified as significant by original PC-TraFF algorithm for all five BRC-subtypes but were determined as specific only in certain subtypes.

**TFBS pair**	**Specific for subtype(s)**	**Pairs documentation**
V$CETS1P54_01 - V$ETS_Q4	*ErbB2 over-expressing, Luminal A*	BioGRID, TransCompel®
V$MYCMAX_B - V$E2F_Q3_01	*Basal-like*	TransCompel®
V$CEBPB_02 - V$STAT6_01	*Luminal A, Luminal B, Basal-like*	TransCompel®
V$STAT6_01 - V$HMGIY_Q6	*Normal-like*	-
V$CETS1P54_01 - V$NFKB_Q6	*Luminal A, Normal-like, Basal-like*	TransCompel®
V$AP1_Q2_01 - V$AP1_Q4_01	*Luminal A, Luminal B, ErbB2 over-expressing*	BioGRID, TransCompel®

*The last column indicates the databases that document the evidence for these pairs. For this purpose, we used TRANSCompel® (Kel-Margoulis et al., 2002) and BioGRID interaction database (Chatr-aryamontri et al., 2014), which contain experimentally proven pairs*.

**Figure 4:**
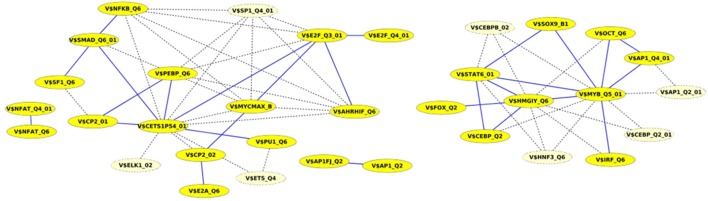
Cooperation network according to PC-TraFF significant TFBS pairs for *Luminal A* gene set. The nodes represent TFBSs identified by the indicated PWMs. Edges represent their potential cooperation based on observed co-occurrences. After applying our extension approach: while blue edges correspond to the sequence-set specific cooperations (α = 0.2), the common cooperations are shown by dashed lines. The nodes with light yellow color indicate TFBSs that are involved in common TF cooperations, but not in the specific pairs.

Finally, we built up cooperation networks based on the significant TFBS pairs, where the nodes refer to TFBSs and edges to predicted co-occurrences and thus, to cooperations between them, in order to demonstrate in an exemplary way the comparative analysis between the results of our extension approach and those of the original PC-TraFF algorithm. The cooperation network based on PC-TraFF significant TFBS pairs for *Luminal A* subtype (see Figure 4) consists of 33 nodes and 62 edges. Reducing the network by only considering sequence-set TFBS pairs results in the elimination of 7 nodes and 35 edges. Consequently, the remaining part of the network is built up of 26 nodes with their 27 sequence-set specific cooperations (edges). It is remarkable that some TFBSs that serve as hubs in the original network are still hub nodes in the reduced network but show a lower number of neighboring nodes (e.g., V$CETS1P54_01, V$MYB_Q5_01, and V$HMGIY_Q6). On the other side, there are some highly connected nodes of the original network that are missing in the specific pair network. For example the degree of V$NFKB_Q6 or V$AHRIF_Q6 decreases from six neighbors to one neighbor and V$SP1_Q4_01 is totally missing in the network of specific pairs. The node representing the binding site V$SMAD_Q6_01 lost just one of its neighbors in this network and thereby, it is among the 25% nodes of highest degree.

**Figure 5:**
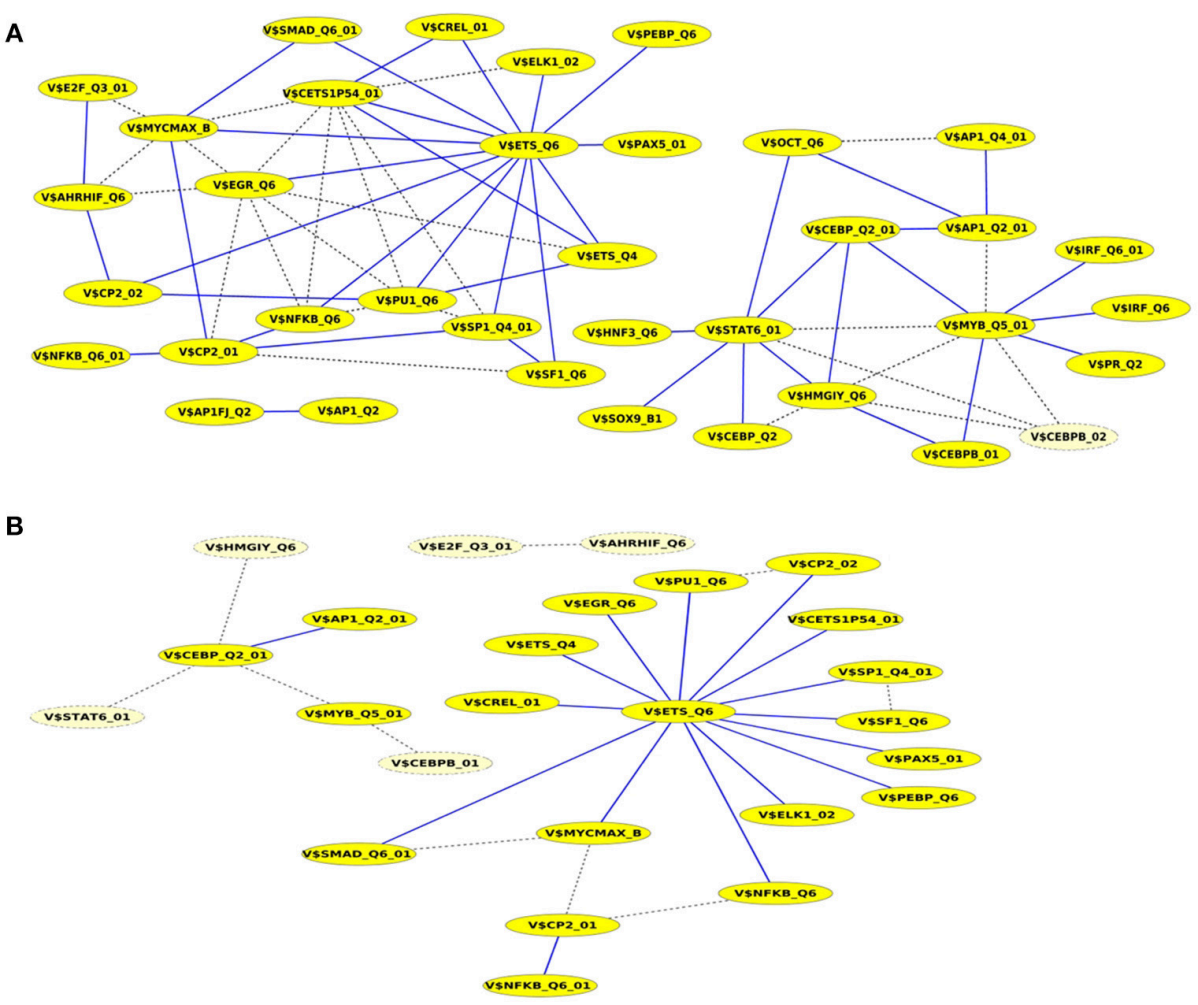
Cooperation network according to PC-TraFF significant TFBS pairs for *Basal-like* gene set. The nodes represent TFBSs identified by the indicated PWMs. Edges represent their potential cooperation based on observed co-occurrences. After applying our extension approach: while blue edges correspond to the sequence-set specific cooperations for **(A)** α = 0.2 and **(B)** α = 0.5, the common (generally important) cooperations are shown by dashed lines. The nodes with light yellow color indicate TFBSs that are involved in common TF cooperations, but not in the specific pairs.

A closer look at the cooperation network of significant TFBS pairs identified for the *Basal-like* data set discloses that 43 out of 68 significant pairs have been assigned to be sequence-set specific based on our extension approach with a scaling factor α = 0.2 (see Figure 5A). Setting α = 0.5 for this analysis leads to elimination of the vast majority of the pairs and consequently 16 pairs have been determined to be specific in the promoter sequences of *Basal-like* subtype (see Figure 5B). A comparison between cooperation networks of *Luminal A* and *Basal-like* subtypes suggests that by considering the same scaling factor our extension approach has more influence on significant pairs found for *Luminal A* data set than those found for *Basal-like* data set. The reason for this finding might be that *Basal-like* data set is more specific than *Luminal A* data set regarding to transcriptional regulation. Thus, the level of background co-occurrence of TFBSs resulting from common regulatory programs seems to be remarkable higher in *Luminal A* data set than those of *Basal-like* data set.

## 3. Methods

### 3.1. Data sets

In order to assess the effectiveness of our approach and to present a detailed comparison with the results of original PC-TraFF algorithm, we analyzed in this study the data sets that have already been reported in Meckbach et al. (2015). The first data set is a simulation data set consisting of 200 sequences with the length of 1000 bps. Each of these sequences contains artificially inserted binding site pairs (V$IRF1_01 - V$USF_01) for the cooperation between transcription factors IRF1 and USF1 with a minimal distance of 5 bp and a maximal distance of 20 bp. For the two inserted binding sites we used the consensus sequences given by the position weight matrices V$IRF1_01 and V$USF_01, respectively.

The second data set is a breast cancer (BRC) gene set determined by Sorlie et al. (2003) and taken from Joshi et al. (2012). The genes have been identified based on their differential mRNA expression behavior in cancer cells and are grouped according to their expression pattern into the five molecular breast cancer-associated subtypes: Luminal A, Luminal B, Normal-like, ErbB2 over-expressing and Basal-like using hierarchical clustering (Sorlie et al., 2003). Our analysis is based on the promoter sequences of the associated genes. The number of genes as well as their corresponding promoter sequences (−500 bp to +100 bp relative to the transcription start site defined by Joshi et al. (2012) in each subtype are given in Table 4. It can be seen that the BRC-subtype data sets differ in the number of genes and consequently in the number of promoter sequences. For example, *Luminal A* gene set appears to be the largest set by consisting of 86 promoter sequences and in turn, the set *ErbB2 over-expressing* is the smallest sequence set by owning 15 promoter sequences (see Table 4). Such differences are important and make it possible to demonstrate the functionality of our extension approach for different sequence-set sizes.

**Table 4:** The number of genes and promoter sequences for the BRC-associated subtypes.

**BRC subtypes**	**Number of genes**	**Number of promoter sequences**
*Luminal A*	78	86
*Luminal B*	55	57
*Normal-like*	23	27
*Basal-like*	28	31
*ErbB2 over-expressing*	13	15

The Methods section of this study comprises two main parts. First, we review our previous work PC-TraFF (Meckbach et al., 2015) so that the readers have sufficient background information to understand the proposed extension in the PC-TraFF workflow. After that, we present our proposed extension approach for the separation of sequence-set specific TF cooperations from common (generally important) ones.

### Previous work: introduction to PC-TraFF

PC-TraFF is an information theory based method that uses the pointwise mutual information (ℙ𝕄𝕀) for the identification of potentially cooperating transcription factors according to their binding site pattern in a set of sequences. The algorithm of PC-TraFF comprises six phases and provides for each TFBS-pair *t*_*a*_ and *t*_*b*_ a ℙ𝕄𝕀_*pc*_(*t*_*a*_, *t*_*b*_)-value based on their distances and frequencies in the sequences, under study.

The overall workflow of PC-TraFF can be briefly given as:

#### 3.1.1. Phase 1: construction and filtering of the TFBS-sequence matrix

In the first step we predict all transcription factor binding sites (TFBSs) in a set of sequences by applying Match™ program (Kel et al., 2003) using the profile parameters and the position weight matrix (PWM) library specified in Deyneko et al. (2013). The PWMs are taken from TRANSFAC database (Wingender, 2008).

**Figure 6:**
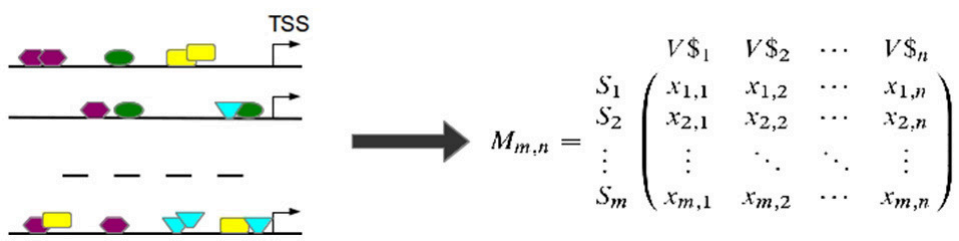
TFBSs are identified for each sequence in the set (left). Afterwards, the TFBS frequencies are stored in a TFBS-sequence matrix 𝕄 where an entry *x*_*i,j*_ is the number of occurrences of TFBS *t*_*j*_ in sequence *s*_*i*_. (TSS stands for “transcription start site”).

Based on the observed frequencies of TFBSs in the sequences under study a TFBS-sequence matrix 𝕄 is constructed (see Figure 6). In 𝕄, the row-names are presented by the IDs of the sequences and columns refer to the names of PWMs used in Match™ algorithm for the prediction of putative TFBSs. An entry *x*_*i, j*_ in 𝕄 is the frequency of a putative TFBS *t*_*j*_ (*j* = 1, .., *n*, where *n* is the number of PWMs) identified by PWM *j* in sequence *s*_*i*_ (*i* = 1, …, *m*, where *m* is the number of sequences under study). After that, columns of 𝕄 are filtered in order to reduce the effect of highly over- or underrepresented TFBSs.

#### 3.1.2. Phase 2: identification of important TFBSs in each sequence

In order to identify important TFBSs for each sequence, we calculate the pointwise mutual information ℙ𝕄𝕀(*s*_*i*_; *t*_*j*_) for each sequence *s*_*i*_ and TFBS *t*_*j*_ pair based on the frequencies of observed TFBSs in each sequence.

$$\begin{array}{l} \mathbb{P}𝕄𝕀\left(s_{i};t_{j}\right)=log_{2}\frac{p\left(s_{i},t_{j}\right)}{p\left(s_{i}\right)p\left(t_{j}\right)}, \end{array}$$

where *p*(*s*_*i*_, *t*_*j*_) is the probability of a TFBS *t*_*j*_ to occur in sequence *s*_*i*_. It is calculated as

(1) $$\begin{array}{l} p\left(s_{i},t_{j}\right)=\frac{f_{ij}}{\sum_{i\;=\;1}^{m}\sum_{j\;=\;1}^{n}f_{ij}} \end{array}$$

where *f*_*ij*_ is the frequency of TFBS *t*_*j*_ in sequence *s*_*i*_. *p*(*s*_*i*_) and *p*(*t*_*j*_) are the marginal probabilities and are calculated as

(2) $$\begin{array}{l} p\left(s_{i}\right)=\frac{\sum_{j=1}^{n}f_{ij}}{\sum_{i\;=\;1}^{m}\sum_{j\;=\;1}^{n}f_{ij}} \end{array}$$

A TFBS *t*_*j*_ is regarded to be important for sequence *s*_*i*_ if the corresponding ℙ𝕄𝕀(*s*_*i*_, *t*_*j*_) > 0. In the following analysis steps, for each sequence only the important TFBSs are considered.

#### 3.1.3. Phase 3: filter to avoid overlaps

**Figure 7:**
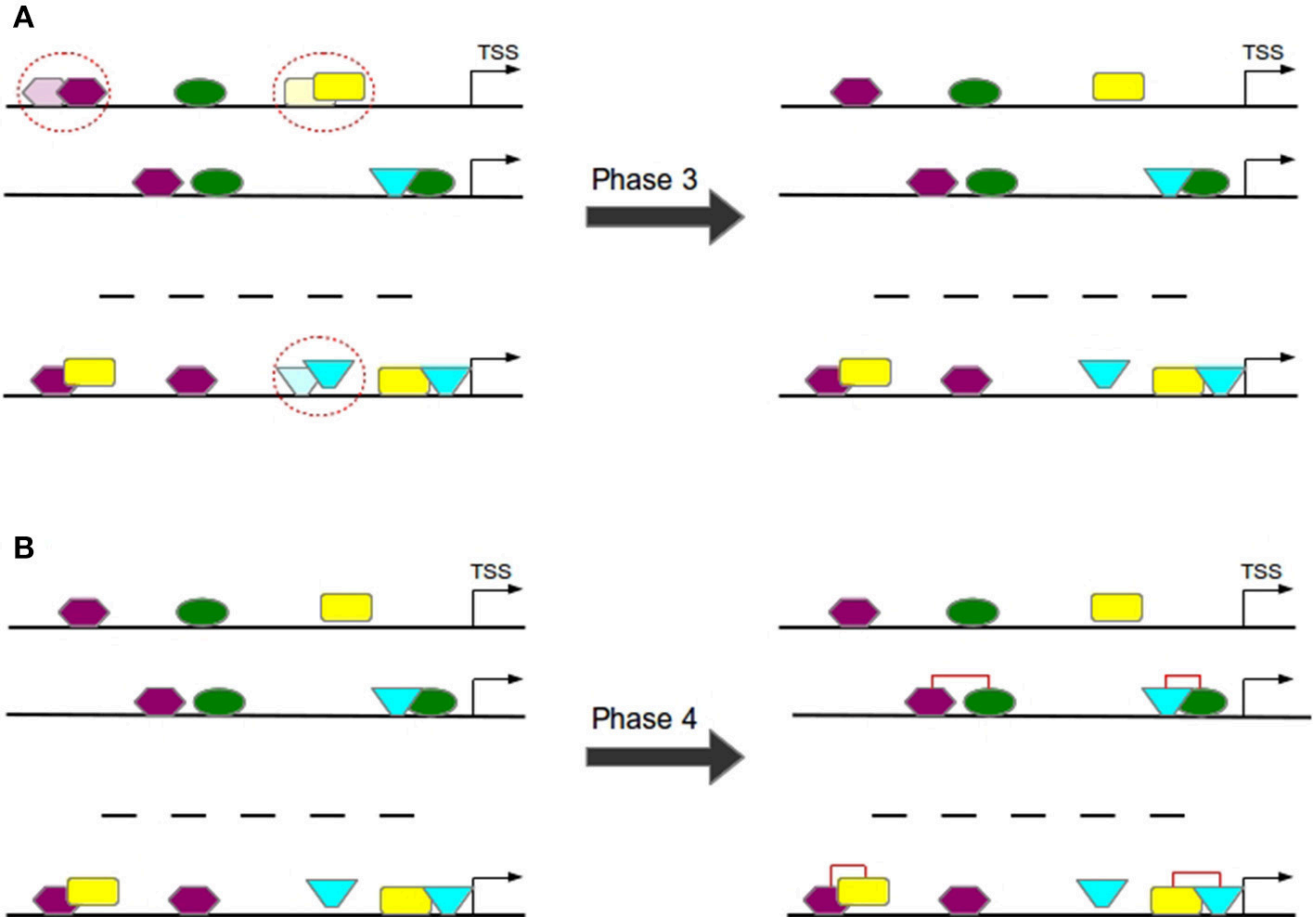
In Phase 3 overlapping TFBSs of the same type are filtered by removing that TFBS having a larger distance to TSS **(A)**. In Phase 4 TFBS pairs are formed according to the distance of their centers **(B)**.

Overlapping TFBSs of the same type are filtered in a way that the TFBS survives which is closer to TSS in order to avoid the overestimation of these repetitive binding sites (see Figure 7A) and thereby to consider only these TFBSs that appear to be more functional (Whitfield et al., 2012).

#### 3.1.4. Phase 4: construction of TFBS pairs

TFBS pairs are identified according to the distance of their centers (see Figure 7B). Two TFBSs can form a pair if their distance satisfies the pre-defined minimal and maximal thresholds.

#### 3.1.5. Phase 5: weighted cumulative pointwise mutual information

The weighted cumulative pointwise mutual information ℙ𝕄𝕀_*pc*_(*t*_*a*_; *t*_*b*_) of two putative TFBSs *t*_*a*_ and *t*_*b*_ is calculated as follows:

(3) $$\begin{array}{l} {\mathbb{P}𝕄𝕀}_{pc}\left(t_{a};t_{b}\right)=\underset{s\in S}{\sum }w_{s}\cdot p\left(t_{a},t_{b}\right)\cdot log_{2}\frac{p\left(t_{a},t_{b}\right)}{p\left(t_{a}\right)\cdot p\left(t_{b}\right)}, \end{array}$$

where *p*(*t*_*a*_, *t*_*b*_), *p*(*t*_*a*_) and *p*(*t*_*b*_) are the joint and marginal probabilities of TFBSs *t*_*a*_ and *t*_*b*_, respectively. Further, *w*_*s*_ refers to the weight of a sequence *s* and is calculated based on the number of TFBS pairs *N*_*s*_ in *s* divided by the total number of TFBS pairs in the entire set of sequences *S*.

(4) $$\begin{array}{l} w_{s}=\frac{N_{s}}{\sum_{s_{i}\in S}N_{s_{i}}} \end{array}$$

#### 3.1.6. Phase 6: background noise reduction of TFBSs using average product correction

To this end, using the average product correction (APC) theorem proposed by Dunn et al. (2008), the ℙ𝕄𝕀_*pc*_(*t*_*a*_; *t*_*b*_) scores have been adjusted:

(5) $${\mathbb{P}𝕄𝕀}_{pc}^{APC}(t_{a};t_{b})={\mathbb{P}𝕄𝕀}_{pc}(t_{a};t_{b})-\frac{{\mathbb{P}𝕄𝕀}_{pc}(t_{a};\bar{t_{x}})\cdot {\mathbb{P}𝕄𝕀}_{pc}(t_{b};\bar{t_{x})}}{\bar{{\mathbb{P}𝕄𝕀}_{pc}}}$$

where ${\mathbb{P}𝕄𝕀}_{pc}\left(t_{a};\bar{t_{x}}\right)$ is the mean ℙ𝕄𝕀_*pc*_ of *t*_*a*_ to all other TFBSs in the sequences, and $\bar{{\mathbb{P}𝕄𝕀}_{pc}}$ is the mean ℙ𝕄𝕀_*pc*_ value over all TFBS pairs.

The resulting ${\mathbb{P}𝕄𝕀}_{pc}^{APC}$ values are transformed into z-scores and only those pairs are considered to be significant that have a z-score ≥3.

### Separation of sequence set specific TF cooperations from the common ones

According to their TFBS motifs, some TF cooperations are noticeable sensitive to the context of nucleotides - regarding the order and positions of nucleotides in sequences - in comparison to common TF cooperations, which are often found as significant for different sequence sets.

In order to separate such sequence-set specific significant TFBS pairs from the common (general important) significant pairs, we propose the following approach: The uShuffle algorithm (Jiang et al., 2008) is used to shuffle the nucleotides within each sequence by setting k-mers' size = 3. Thereby, not only the single nucleotide counts of each sequence are maintained but also the triplet counts and thus, the core of TFBSs. By repeating this shuffling process several times, a sufficient number of randomly generated sequence sets (e.g., 1000) is created.

Second, employing the Match™ algorithm for each set of shuffled sequences, the putative binding sites of TFs in these sequences are predicted. Third, applying PC-TraFF algorithm, new ℙ𝕄𝕀_*pc*_-values for every TFBS pair in each randomly generated sequence set are calculated. Fourth, based on these ℙ𝕄𝕀_*pc*_-values of each pair *t*_*a*_ and *t*_*b*_, we define the average ℙ𝕄𝕀-value, *AVG*(ℙ𝕄𝕀(*t*_*a*_; *t*_*b*_)) as

(6) $$\begin{array}{l} AVG\left(\mathbb{P}𝕄𝕀\left(t_{a};t_{b}\right)\right)=\frac{1}{l}\overset{l}{\underset{i=1}{\sum }}{\mathbb{P}𝕄𝕀}_{pc}^{APC}{\left(t_{a};t_{b}\right)}_{i}, \end{array}$$

where *l* is the number of randomly generated sequence sets.

After that, the *AVG*(ℙ𝕄𝕀(*t*_*a*_; *t*_*b*_))-value of binding sites *t*_*a*_ and *t*_*b*_ is subtracted from their initial significant ${\mathbb{P}𝕄𝕀}_{pc}^{APC}\left(t_{a};t_{b}\right)$ -value as

(7) $$\begin{array}{l} {\mathbb{P}𝕄𝕀}^{specific}\left(t_{a};t_{b}\right)={\mathbb{P}𝕄𝕀}_{pc}^{APC}\left(t_{a};t_{b}\right)-[\left(1+\alpha \right)\times AVG\left(\mathbb{P}𝕄𝕀\left(t_{a};t_{b}\right)\right)], \end{array}$$

where α ∈ [−1, +1] is a preassigned real number for monitoring the influcene of this process on the significant TFBS pairs. It can easily be seen that α = −1 results in the original PC-TraFF analysis. By setting α =0 the average *AVG*(ℙ𝕄𝕀(*t*_*a*_; *t*_*b*_)) is subtracted from the original ${\mathbb{P}𝕄𝕀}_{pc}^{APC}\left(t_{a};t_{b}\right)$ value whereas an α ≥ 0 leads to a stronger effect of the subtraction and thus, a more strict selection process. However, for the proper application of this process the determination of an upper bound for α is crucial in order to avoid the overestimation of the efficacy of *AVG*(ℙ𝕄𝕀(*t*_*a*_; *t*_*b*_))-values (background level) on the separation of sequence-set specific pairs from common ones. By systematically analyzing different values, we established that +1 is the most convenient upper bound for α.

A positive ${\mathbb{P}𝕄𝕀}^{specific}\left(t_{a};t_{b}\right)$-value of binding sites *t*_*a*_ and *t*_*b*_ identified in the promoter sequences of a certain sequence set suggests that the binding of the related TF pair is strongly sequence context dependent. In contrast, a ${\mathbb{P}𝕄𝕀}^{specific}\left(t_{a};t_{b}\right)$-value ≤ 0 indicates that the cooperations of corresponding TFs could have a general importance for the controlling of genetic programs.

## 4. Conclusions

Depending on their biological functions as well as cellular context, TFs specify the selection of cooperation partners in many ways for different cell types. However, the existing algorithms often focus on the identification of all predictable TF cooperations without distinguishing between sequence-set specific and common, i.e., ubiquitously occurring TF cooperations. To address this limitation, we propose in this study an approach that extends our previous method PC-TraFF in order to assign its predictions into two main categories: sequence-set specific and common (generally important) ones. For this aim, we estimated the background co-occurrence of any TF pair by preserving the nucleotide composition and the core of TFBS motifs in the sequences of interest. To maintain the core of TFBS motifs, we set the *k-mers*'size = 3 in the randomly shuffled new sets of sequences. It can be seen that, while an increase in *k-mers*'size could lead to increment of background co-occurrence of TFBSs, a decrease in *k-mers*'size could in turn result in the reduction of background level of TF pairs. In order to assess the effectiveness of our extension approach, we analyzed promoter sequences of five different breast cancer-associated subtypes. The results show that the cooperating pairs identified by original PC-TraFF algorithm were considerably overlapping between the subtypes. Applying our extension approach, we could successfully separate sequence-set specific pairs from common ones and thereby reducing the number of overlapping pairs. Further, when we applied our extension approach of the original PC-TraFF algorithm to a simulation data set with varying α-values and, thus, different background levels, we could demonstrate that the cooperating TF pair was consistently identified as a sequence-set specific pair. The scaling parameter α is useful to extend or reduce the level of the subtracted background. Thereby, the influence of α itself is not linear but highly depending on the sequence set and thus on the respective background. Starting with an α-value of 0.2 we recommend to slightly increase α in order to assess the effect of α on the given data set and in doing so, to get the desired ratio between sensitivity and specificity. In summary, the proposed extension approach can successfully be applied for the distinction of sequence-set specific TF cooperations from common ones which are identified as generally important for different data sets.

## Availability of data and algorithm

The extension of PC-TraFF is freely accessible at http://pctraffpro.bioinf.med.uni-goettingen.de/. All data sets and results of this paper are available from the corresponding author on request.

## Author contributions

CM and MG developed the model and conducted computational analyses. EW interpreted the results and adjusted the model together with CM and MG. CM and MG conceived of and managed the project and wrote the final version of the manuscript. All authors read and approved the final manuscript.

### Conflict of interest statement

EW is head of geneXplain GmbH, the company that maintains and distributes the TRANSFAC database. The other authors declares that the research was conducted in the absence of any commercial or financial relationships that could be construed as a potential conflict of interest.
